# The effect of external beam radiotherapy volume on locoregional control in patients with locoregionally advanced or recurrent nonanaplastic thyroid cancer

**DOI:** 10.1186/1748-717X-5-69

**Published:** 2010-08-06

**Authors:** Tae Hyun Kim, Ki-Wook Chung, You Jin Lee, Chan Sung Park, Eun Kyung Lee, Tae Sung Kim, Seok Ki Kim, Yoo Seok Jung, Jun Sun Ryu, Sang Soo Kim, Kwan Ho Cho, Kyung Hwan Shin

**Affiliations:** 1 Center for Thyroid Cancer, Research Institute and Hospital, National Cancer Center, Goyang, Korea

## Abstract

**Purpose:**

We evaluated outcomes of patients treated with external beam radiotherapy (EBRT) for locoregionally advanced or recurrent nonanaplastic thyroid cancer and analyzed the effect of EBRT volume on locoregional control.

**Methods:**

This study included 23 patients with locoregionally advanced or recurrent nonanaplastic thyroid cancer who were treated with EBRT. Two different EBRT target volumes were executed as follows: 1) limited field (LF, n = 11) included the primary (involved lobe) or recurrent tumor bed and the positive nodal area; 2) elective field (EF, n = 12) included the primary (involved lobe) or recurrent tumor bed and the regional nodal areas in the cervical neck and upper mediastinum. Clinical parameters, such as gender, age, histologic type, recurrence, stage, thyroglobulin level, postoperative residuum, radioiodine treatment, and EBRT volume were analyzed to identify prognostic factors associated with locoregional control.

**Results:**

There were no significant differences in the clinical parameter distributions between the LF and EF groups. In the LF group, six (55%) patients developed locoregional recurrence and three (27%) developed distant metastasis. In the EF group, one (8%) patient developed locoregional recurrence and one (8%) developed a distant metastasis. There was a significant difference in locoregional control rate at 5 years in the LF and EF groups (40% vs. 89%, *p *= 0.041). There were no significant differences in incidences of acute and late toxicities between two groups (*p >*0.05).

**Conclusions:**

EBRT with EF provided significantly better locoregional control than that of LF; however, further larger scaled studies are warranted.

## Introduction

Surgical resection, radioactive iodine treatment (RAI), and thyroid-stimulating hormone suppression are considered as standard treatments for nonanaplastic thyroid cancer. However, the role of external beam radiotherapy (EBRT) remains controversial. Despite conflicting data [1-4], a number of retrospective studies have demonstrated that EBRT potentially improves locoregional control in patients with nonanaplastic thyroid cancer who have high risk features for locoregional recurrence, such as pT4, lymph node involvement, micro-/macroscopic positive surgical margins, extensive extrathyroidal or extranodal extension at recurrence, or RAI-resistant recurrent disease [5-16]. To date, the current indication, radiation dose, and irradiated volume of EBRT have largely been determined from retrospective data.

Theoretically, EBRT is directed to the thyroid bed and draining lymphatics in the cervical neck and upper mediastinum to achieve locoregional control and it is recommended [17,18]. But, in clinical practice, EBRT volume has varied among and within studies from the thyroid bed only to the thyroid bed and regional nodal area in the cervical neck and upper mediastinum. There are also differences in the regional nodal areas that need to be irradiated. The superior and inferior borders of EBRT volume have been variously defined as follows: the tip of the mastoid process, the hyoid bone level and upper level of the thyroid bed; below the suprasternal notch and the level of the carina, respectively. These large differences in EBRT volume among those studies make interpretation difficult and, thus, it is needed to evaluate the effect of EBRT volume on locoregional control in nonanaplatic thyroid cancer patients. Additionally, it is important for patients with nonanaplastic thyroid cancer to undergo EBRT in clinical practice. Therefore, we evaluated our institutional outcomes of patients treated with EBRT for locally advanced or recurrent nonanaplastic thyroid cancer and analyzed the effect of EBRT volume on locoregional control.

## Methods

### Patients

Between February 2004 and February 2008, 25 patients with locoregionally advanced or recurrent nonanaplastic thyroid cancer were treated with EBRT to the primary or recurrent site and/or regional nodal area at the National Cancer Center (NCC), Korea. Of these, two patients with medullary carcinoma were excluded. This retrospective study was performed in accordance with the guidelines of the Institutional Review Board of the NCC. The patients consisted of nineteen (83%) women and four (17%) men ranging in age from 37 to 76 years (median, 64 years) (Table 1). All patients had a histologically confirmed thyroid carcinoma; nineteen had papillary carcinoma, and four had poorly differentiated carcinoma, which were defined based on ≥ 5 mitoses/10 high power microscopic fields (400×) and/or tumor necrosis[19,20]. The stage of all patients were determined using the American Joint Committee on Cancer (AJCC) seventh edition staging system [21] and had more than one of the high risk clinicopathologic features, such as the presence of microscopic or gross residual disease, soft tissue extension of the primary tumor (T4), or nodal metastasis (N+). Fifteen (65%) patients had locoregionally recurrent disease. Five (22%) patients had a distant metastasis which was controlled with stable disease at pre-EBRT and these patients' histologic type was all papillary.

**Table 1:** Patient characteristics

Patient Characteristics		Distribution, n (%)
Gender	Female	19 (83)
	Male	4 (17)
Age (years)	Median (range)	64 (37-76)
	< 45	2 (8)
	45 - 64	10 (44)
	≥ 65	11 (48)
KPS	90	10 (43)
	80	13 (57)
Histologic type	Papillary	19 (83)
	Poorly differentiated	4 (17)
Recurrence	Primary tumor	8 (35)
	Recurrent tumor	15 (65)
T stage at initial diagnosis	T1-2	1 (4)
	T3	14 (61)
	T4	8 (35)
N stage at initial diagnosis	N0	4 (17)
	N1a	10 (44)
	N1b	9 (39)
AJCC Stage at initial diagnosis	I-II	3 (12)
	III	10 (44)
	IVA	10 (44)
T stage at pre-EBRT	T0-2	3 (13)
	T3	3 (13)
	T4	17 (74)
N stage at pre-EBRT	N0	3 (13)
	N1a	4 (17)
	N1b	16 (70)
AJCC Stage at pre-EBRT	I-III	3 (13)
	IVA	14 (61)
	IVB	1 (4)
	IVC	5 (22)
Tg* level(ng/mL)	Median (range)	10 (0.2-1411)
	< 10	11 (48)
	≥ 10	12 (52)
Thyroid surgery	Subtotal thyroidectomy	4 (17)
	Total thyroidectomy	19 (83)
Lymph node surgery	CND	6 (26)
	CND+Unilateral SND/MRND	14 (61)
	CND+Bilateral SND/MRND	3 (13)
Postoperative residuum	No	3 (13)
	Microscopic	2 (9)
	Macroscopic	18 (78)
Radioiodine treatment	No	4 (17)
	Yes	19 (83)
EBRT volume	Limited field	11 (48)
	Elective field	12 (52)
EBRT dose (EQD_2_, Gy)	Median (range)	62.5 (60-69)

Abbreviation: KPS = Karnofsky performance status; AJCC, American Joint Committee on Cancer; EBRT, external beam radiotherapy; Tg* = pre-EBRT serum thyroglobulin; CND = central compartmental neck dissection; SND = selective neck dissection; MRND = modified radical neck dissection; EQD_2_, biologically equivalent dose in 2 Gy fractions using a linear quadratic model (α/β ratio for acute effects on normal tissues and tumors = 10).

### Treatment

Before surgery, a complete medical history, physical examination, routine blood counts, serum chemistry, and imaging studies including neck ultrasound (US), computed tomography (CT) and/or magnetic resonance imaging (MRI) were performed to accurately evaluate the extent of the primary or recurrent tumor and regional lymph nodes and to assess the resectability of the disease. Of the 23 patients, 19 (83%) underwent surgery before EBRT. Eighteen (78%) patients, including 4 with unresectable disease, had gross residual disease, two (9%) patients had microscopic residual disease, and three (13%) patients had no residual disease. After surgery, nineteen (93%) patients received a radioactive iodine (RAI) at a dose of 150 (n = 13) or 200 mCi (n = 6) and four patients, whose composed of three poorly differentiated and one papillary carcinoma patients, did not received a RAI. After surgery and/or RAI, all patients were treated with EBRT.

All patients underwent CT simulation for EBRT in the treatment position, which was generally supine, and they were immobilized from the head to the shoulders using a thermoplastic mask. Target volumes and the normal critical tissues, such as the spinal cord, parotid glands, larynx, and esophagus, were delineated on the treatment planning CT images. Two different EBRT target volumes, according to the patient and physician's preferences (e.g., age, history of recurrence, anxiety, and compliance for EBRT), were executed as follows: 1) In limited field group (LF, n = 11), the median total dose of 62.5 Gy (range, 62.5-67.7 Gy) was delivered to the primary (involved lobe) or recurrent tumor bed and the positive nodal area; 2) in elective field group (EF, n = 12), the median dose of 50 Gy (range, 44-54 Gy) was delivered to the initial target volume [primary (involved lobe) or recurrent tumor bed and regional nodal area in the cervical neck and upper mediastinum] and then median total dose of 62.5 Gy (range, 60-69 Gy) was delivered to boost target volume (tumor bed and positive nodal area). Radiation dose values for all patients were converted to the equivalent dose in 2 Gy fractions using a linear quadratic model, and the α/β ratio was 10 for the acute effects on normal tissues and tumors [22], because the thirteen patients received a daily fraction size of 2.5 Gy, four patients received 2 Gy and the three patients received 1.8 Gy. The planning for the EBRT was done using 3-dimensional conformal radiotherapy (3DCRT) techniques in 17 (74%) patients and intensity modulated radiotherapy (IMRT) techniques in 6 (26%) patients who were difficult to obtain the adequate target coverage within the normal tissues constraints with 3DCRT. When the 3DCRT was used for treating the initial target volume in the EF group, anterior-posterior parallel pair technique, a dominant weighting (3-4:1) to anterior beam as compared to posterior beam together with posterior midline shielding after 36-40Gy, was primarily used. During planning, the mean dose to the parotid gland was constrained to < 26 Gy, and the total doses to the spinal cord, larynx, and esophagus were constrained to < 45 Gy, < 60 Gy, and < 60 Gy, respectively.

### Evaluation of Locoregional Recurrence and Follow-up

Patient follow-up by a radiation oncologist, endocrinologist, or thyroid surgeon was performed every 3 months for the first 2 post-operative years, then every 6 months for up to 5 years, and yearly thereafter. For all patients, follow-up evaluations included a physical examination, complete blood count, liver function test, serum thyroglobulin (Tg) level, neck US and/or CT, ^131^I whole body scan, and other radiologic studies, if necessary. Recurrence was demonstrated pathologically by surgical resection, biopsy, or cytology, and/or radiological findings, which increased in size over time; locoregional recurrence was defined as tumor recurrence within a tumor bed or nodal areas in the cervical neck and the upper mediastinum, and distant metastasis was defined as any recurrence outside of the cervical neck and the upper mediastinum. Locoregional recurrence was subdivided into infield and outfield recurrences; infield recurrence was defined as locoregional recurrence within the RT field; and outfield recurrence was defined as locoregional recurrence outside of the RT field.

### Statistical Analysis

Clinical parameters, such as gender, age (<45, 45-64, and ≥ 65 years), Karnofsky performance status (90 vs. 80), histologic type (papillary vs. poorly differentiated), recurrence (primary tumor vs. recurrent tumor), T stage (T1-2, T3, and T4), N stage (N0, N1a, and N1b), AJCC stage at diagnosis (I-III vs. IVA) and at pre-EBRT(I-III, IVA-B, and IVC), Tg level (< 10 ng/mL vs. ≥ 10 ng/mL), thyroid surgery (subtotal vs. total thyroidectomy), postoperative residuum (R0-1 vs. R2), radioiodine treatment (no vs. yes), EBRT techniques (3DCRT vs. IMRT), and EBRT volume (LF vs. EF) were considered categorical variables. Fisher's exact test was used to compare the distribution of clinical parameters between the LF and EF groups. Locoregional control, disease-free survival, and overall survival were defined as the interval from the commencement date of EBRT to the date of locoregional recurrence detection, any recurrence detection, and death, respectively. Local control, disease-free survival, and overall survival probability were calculated using the Kaplan-Meier method. The analysis for effect of EBRT volume on locoregional control was performed by comparing locoregional control rates using the log rank test. All statistical tests were two-sided and performed using STATA software (version 9.0; Stata Corp., College Station, TX, USA). A *p *value of < 0.05 was considered statistically significant.

## Results

At last follow-up, 19 (83%) patients were alive and 15 (65%) remained free of disease. Of the 23 patients, locoregional recurrence developed in seven (30%) and new distant metastasis in four (17%), of which two patients had simultaneous locoregional recurrence, and new distant metastasis. The site of newly developed distant metastasis included lung (n = 2), bone (n = 1), and brain (n = 1). All four patients with newly developed distant metastasis had received a RAI prior to EBRT and had papillary histology. For all patients, overall survival, disease-free survival, and the locoregional control rate at 5 years were 75%, 57%, and 61%, respectively. The median time to locoregional recurrence was 25.3 months (range, 15.5-36.7 months), and the median follow-up time for all patients and for surviving patients was 41.1 months (range, 8.3-69.4 months) and 43.3 months (range, 24-69.4 months), respectively.

**Figure 1:**
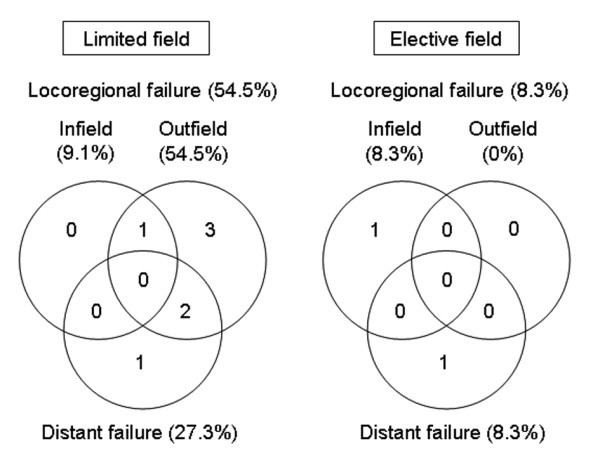
**Patterns of treatment failure in the limited field and elective field groups**.

**Figure 2:**
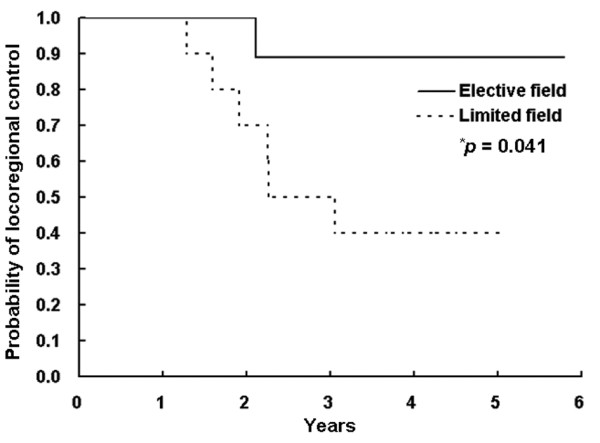
**Locoregional control curves in the limited field and elective field groups**. *log-rank test.

The comparison of clinical parameters between the LF and EF groups is summarized in Table 2. There were no significant differences in the distributions of clinical parameters between the groups. Figure 1 illustrates the patterns of treatment failure in each group. In the LF group (n = 11), six (55%) patients developed locoregional recurrence and three (27%) developed new distant metastases, of which two had simultaneous locoregional recurrence and new distant metastasis. Of six patients who had locoregional recurrence, six (100%) had outfield failure and one (17%) had infield failure, of which one patient had simultaneous infield and outfield failure. In the EF group (n = 12), one (8%) patient developed locoregional recurrence and one (8%) developed new distant metastasis. The failure site of one patient who had locoregional recurrence was infield. There were no significant differences in overall survival and disease-free survival rate at 5 years in the LF and EF groups (67% vs. 88%, *p *= 0.475; 40% vs. 78%, *p *= 0.113, respectively), whereas there was a significant difference in locoregional control rate at 5 years in the LF and EF groups (40% vs. 89%, *p *= 0.041) (Figure 2). The infield control rate at 5 years in the LF and EF groups was not significantly different (88% vs. 89%, *p *= 0.779). The incidences of acute and late toxicities between LF and EF field groups are summarized in Tables 3. There were no significant differences in incidences of acute and late toxicities between two groups (*p *> 0.05). None of the patients in both groups developed higher than grade 4 acute and chronic toxicities, and none of patients who developed locoregional recurrence were subsequently inoperable due to EBRT.

**Table 2:** Comparison of patient characteristics between the limited field and elective field groups

Patient Characteristics		Limited field	Elective field	*p*-value
			
		(n = 11)	(n = 12)	
Gender	Female	9	10	1.000*
	Male	2	2	
Age (years)	< 45	0	2	0.582*
	45 - 64	5	5	
	≥ 65	6	5	
KPS	90	4	6	0.680*
	80	7	6	
Histologic type	Papillary	11	8	0.093*
	Poorly differentiated	0	4	
Recurrence	Primary tumor	3	5	0.667*
	Recurrent tumor	8	7	
T stage at diagnosis	T1-2	1	0	0.822*
	T3	6	8	
	T4	4	4	
N stage at diagnosis	N0	3	1	0.179*
	N1a	6	4	
	N1b	2	7	
AJCC Stage at diagnosis	I-III	7	6	0.680*
	IVA	4	6	
T stage at pre-EBRT	T0-2	2	2	1.000*
	T3	1	2	
	T4	8	8	
N stage at pre-EBRT	N0	2	1	0.359*
	N1a	3	1	
	N1b	6	10	
AJCC Stage at pre-EBRT	I-III	1	2	0.640*
	IVA-B	7	8	
	IVC	3	2	
Tg level (ng/mL)	Median (range)	13.5 (2.7-449)	10.7 (0.2-1411)	0.257^†^
	Mean ± SD	76.8 ± 137.7	232.3 ± 431.7	
	< 10	5	6	1.000*
	≥ 10	6	6	
Thyroid surgery	Subtotal thyroidectomy	2	2	1.000*
	Total thyroidectomy	9	10	
Lymph node surgery	CND	3	3	1.000*
	CND+Unilateral SND/MRND	7	7	
	CND+Bilateral SND/MRND	1	2	
Postoperative residuum	R0-1	2	3	1.000*
	R2	9	9	
Radioiodine treatment	No	1	3	0.590*
	Yes	10	9	
EBRT dose (EQD_2_, Gy)	Median (range)	62.5 (62.5-67.7)	62.5 (60-69)	0.110^†^
	Mean ± standard deviation	63.3 ± 1.8	65.1 ± 3.1	
EBRT techniques	3DCRT	3	3	1.000*
	IMRT	8	9	

Abbreviation: 3DCRT = 3-dimensional conformal radiotherapy; IMRT = intensity modulated radiotherapy; same as in Table 1.

*Fisher's exact test.

^†^t-test.

**Table 3:** Incidences of acute and late toxicity between the limited field and elective field groups

Toxicity	Limited field (n = 11)	Elective field (n = 12)	*p*-value*
		
	Grade 1/Grade 2/Grade 3 (n)	Grade 1/Grade 2/Grade 3 (n)	
Acute			
Skin	5/6/0	8/4/0	0.414
Mucositis	6/5/0	2/10/0	0.089
Xerostomia	4/0/0	7/2/0	0.140
Dysphagia	4/7/0	3/8/1	1.000
Late			
Skin	7/0/0	6/0/0	0.680
Xerostomia	2/0/0	4/0/0	0.640
Laryngeal edema	2/0/0	3/0/0	1.000

*Fisher's exact test.

Grade determined using National Cancer Institute Common Toxicity Criteria for Events, version 3.0.

## Discussion

The role of EBRT in patients with nonanaplastic thyroid cancer is controversial due to the different perspectives of surgeons, endocrinologists, nuclear medicine specialists, and radiation oncologists, and also because controlled randomized clinical trials have not been conducted. However, a number of studies have supported the use of EBRT in populations with high-risk features for relapse and gross residual disease [5-16]. These studies have demonstrated that patients with pT4, lymph node involvement, micro-/macroscopic positive surgical margins, extensive extrathyroidal or extranodal extension at recurrence, or RAI-resistant recurrent disease might be candidates for EBRT. The American Thyroid Association Guidelines Task Force now recommends EBRT for gross residual disease not amendable to surgery or RAI treatment [23]. On the basis of these studies, nonanaplastic thyroid cancer patients at our institution are selected for EBRT if they have high risk features for locoregional recurrence.

When undergoing EBRT for nonanaplastic thyroid cancer, theoretically, the EBRT volume, directing to the thyroid bed and draining lymphatics in the cervical neck and upper mediastinum, is recommended[17,18]. Recently, Arif et al. showed that all mediastinal recurrences after EBRT occurred in the superior mediastinum and recommended that the target volume should include the thyroid bed, regional neck, and superior mediastinum [24]. However, in clinical practice, in order to decrease the toxicities of EBRT and to escalating the radiation dose for improving the local control, the EBRT volume, such as inclusion and extent of regional nodal areas, for nonanaplastic thyroid cancer has varied tremendously among and within centers[5-16] from the thyroid bed only to the thyroid bed and regional nodal areas. These large differences in EBRT volume could partially explain the differences in the observed locoregional control of disease and, in particular, the treatment-related toxicities [11,25-27]. Therefore, it is required to evaluate the effect of EBRT volume on locoregional control in patients with nonanaplastic thyroid cancer. In this study, we reviewed our institutional outcomes of patients treated with EBRT for locoregionally advanced and recurrent nonanaplastic thyroid cancer to evaluate which EBRT volume, such as LF (primary or recurrent tumor site and positive nodal area) or EF (the primary or recurrent tumor site and regional nodal areas in the cervical neck and the upper mediastinum) should be included

In our study, the locoregional control rate of patients irradiated with EF was significantly better than those irradiated with LF (89% vs. 40%, *p *= 0.041). However, overall survival and disease-free survival of the patients irradiated with EF tended to be higher than those irradiated with LF, but the differences were not significant (88% vs. 67%, *p *= 0.475; 78% vs. 40%, *p *= 0.113, respectively). All patients in the present study had high risk features, such as incomplete surgical resection, extensive soft tissue extension, extranodal extension, poorly differentiated subtypes, or RAI-resistant recurrent disease. In particular, most patients (78%) had gross residual disease; however, the infield control rates at 5 years in the LF and EF groups were 88% and 89%, respectively (*p *= 0.779). The infield (local) control rates in our study are comparable to those of previous studies [5-9,11,12,24,28]. These results suggested that EBRT with LF or EF effectively improved the local control of nonanaplastic thyroid cancer with high risk features and EBRT with EF could more effectively improve not only local control but also locoregional control than EBRT with LF. This implied that EBRT with EF, irradiating to tumor bed and regional lymph node area in the cervical neck and upper mediastinum to eradicate the macro-/microscopic disease, might be beneficial in nonanaplastic thyroid cancer patients with high risk features by improving locoregional control even if local control could be achieved by other treatments, such as EBRT with LF, surgery, and/or RAI. However, the present study had a relatively small sample size (*n *= 23) with a heterogeneous population experiencing different postoperative residual disease including recurrent disease and adjuvant RAI treatment and had relatively short-term outcomes. Therefore, further larger-scale studies should be needed to determine the role of EBRT and the effect of EBRT volume on locoregional control for nonanaplastic thyroid cancer patients with high risk features.

Resistance to routinely performing EBRT for patients with nonanaplastic thyroid cancer occurs principally for several reasons. First, the survival benefit of EBRT for nonanaplastic thyroid cancer is meager. To base a treatment strategy solely on survival data would ignore the problems associated with locoregional recurrence, such as the risk of anaplastic transformation in patients with long-standing locoregional disease, the increased risk of re-operation, the patient's mental anguish, and the considerable time and expense of repeated evaluations. Moreover, the progression of locoregional disease can significantly affect morbidity and quality of life because of the proximity to critical organs, including the esophagus, larynx, and spinal cord. For example, Chow et al. reported that locoregional disease was the major contributing cause of death in patients with locally advanced nonanaplastic thyroid cancer[8]. Therefore, locoregional control is an important endpoint when examining outcomes after EBRT in patients with locoregionally advanced and recurrent nonanaplastic thyroid cancer with and without metastatic disease, and EBRT should be considered for these patients. Second, treatment-related toxicities are important because of the difficulty in delivering an adequate dose of EBRT without exceeding the tolerance of critical organs when using conventional two-dimensional EBRT techniques [11,24,27,29]. With the advent of 3-dimensional (3D) planning systems, 3DCRT and IMRT techniques improve the target coverage within acceptable treatment-related toxicities by limiting the irradiated volume of these normal tissues [11,27,29]. In our study, acute toxicities were self-limiting and none of patients developed higher than grade 3 late toxicities.

## Conclusion

In conclusion, we evaluated the outcomes of EBRT in patients with locoregionally advanced or recurrent nonanaplastic thyroid cancer that had high-risk features for locoregional recurrence and analyzed the effect of EBRT volume on locoregional control. Our study showed that EBRT with EF had significantly better locoregional control than that of LF and suggested that EBRT with EF might benefit in nonanaplastic thyroid cancer patients by improving locoregional control even if local control could be achieved by other treatments. However, this study enrolled a small number of patients and had a relatively short follow-up period. Therefore, further larger scaled studies with a longer follow-up are required to accurately define the effect of EBRT and EBRT volume on locoregional control for locally advanced or recurrent nonanaplastic thyroid cancer. In the meanwhile, we recommend EBRT with EF for patients with locally advanced or recurrent nonanaplastic thyroid cancer.

## Competing interests

The authors declare that they have no competing interests.

## Authors' contributions

THK and KHS are responsible for the study design. THK, KWK, YJL, CSP, TSK, SKK, YSJ and JSR collected the clinical data and drafted the manuscript. THK, KHS, KHC, EKL and SSK revised the manuscript. KWC and YJL collected the pathologic data and analysis THK, KHS, KWC, and YJL were responsible for the treatment and evaluation of the patients. KHS and THK provided oversight of the analysis of data and reviewing of the manuscript. All authors read and approved the final manuscript.
